# Evaluation of NGS-based approaches for SARS-CoV-2 whole genome characterisation

**DOI:** 10.1093/ve/veaa075

**Published:** 2020-10-05

**Authors:** Caroline Charre, Christophe Ginevra, Marina Sabatier, Hadrien Regue, Grégory Destras, Solenne Brun, Gwendolyne Burfin, Caroline Scholtes, Florence Morfin, Martine Valette, Bruno Lina, Antonin Bal, Laurence Josset

**Affiliations:** Laboratoire de Virologie, Institut des Agents Infectieux (IAI), Hospices Civils de Lyon, Groupement Hospitalier Nord, Lyon cedex 4, France; Université de Lyon, Université Claude Bernard Lyon 1 (UCBL1), Lyon, France; Centre de recherche en cancérologie (CRCL), INSERM U1052- 69008, Lyon, France; CIRI, Centre International de Recherche en Infectiologie, (Team pathogenesis of Legionella), Univ Lyon, Inserm, U1111, Université Claude Bernard Lyon 1, CNRS, UMR5308, ENS de Lyon, Lyon 69007, France; Hospices Civils de Lyon, Centre National de Référence des Légionelles, Lyon, France; Laboratoire de Virologie, Institut des Agents Infectieux (IAI), Hospices Civils de Lyon, Groupement Hospitalier Nord, Lyon cedex 4, France; Université de Lyon, Université Claude Bernard Lyon 1 (UCBL1), Lyon, France; Université de Lyon, Virpath, CIRI, INSERM U1111, CNRS UMR5308, ENS Lyon, Université Claude Bernard Lyon 1, Lyon, France; Laboratoire de Virologie, Institut des Agents Infectieux (IAI), Hospices Civils de Lyon, Groupement Hospitalier Nord, Lyon cedex 4, France; Université de Lyon, Université Claude Bernard Lyon 1 (UCBL1), Lyon, France; Laboratoire de Virologie, Institut des Agents Infectieux (IAI), Hospices Civils de Lyon, Groupement Hospitalier Nord, Lyon cedex 4, France; Université de Lyon, Université Claude Bernard Lyon 1 (UCBL1), Lyon, France; Université de Lyon, Virpath, CIRI, INSERM U1111, CNRS UMR5308, ENS Lyon, Université Claude Bernard Lyon 1, Lyon, France; Centre National de Référence France-Sud des Virus des Infections Respiratoires, Hospices Civils de Lyon, Groupement Hospitalier Nord, Lyon cedex 4, France; Laboratoire de Virologie, Institut des Agents Infectieux (IAI), Hospices Civils de Lyon, Groupement Hospitalier Nord, Lyon cedex 4, France; Centre National de Référence France-Sud des Virus des Infections Respiratoires, Hospices Civils de Lyon, Groupement Hospitalier Nord, Lyon cedex 4, France; Laboratoire de Virologie, Institut des Agents Infectieux (IAI), Hospices Civils de Lyon, Groupement Hospitalier Nord, Lyon cedex 4, France; Centre National de Référence France-Sud des Virus des Infections Respiratoires, Hospices Civils de Lyon, Groupement Hospitalier Nord, Lyon cedex 4, France; Laboratoire de Virologie, Institut des Agents Infectieux (IAI), Hospices Civils de Lyon, Groupement Hospitalier Nord, Lyon cedex 4, France; Université de Lyon, Université Claude Bernard Lyon 1 (UCBL1), Lyon, France; Centre de recherche en cancérologie (CRCL), INSERM U1052- 69008, Lyon, France; Laboratoire de Virologie, Institut des Agents Infectieux (IAI), Hospices Civils de Lyon, Groupement Hospitalier Nord, Lyon cedex 4, France; Université de Lyon, Université Claude Bernard Lyon 1 (UCBL1), Lyon, France; Université de Lyon, Virpath, CIRI, INSERM U1111, CNRS UMR5308, ENS Lyon, Université Claude Bernard Lyon 1, Lyon, France; Centre National de Référence France-Sud des Virus des Infections Respiratoires, Hospices Civils de Lyon, Groupement Hospitalier Nord, Lyon cedex 4, France; Laboratoire de Virologie, Institut des Agents Infectieux (IAI), Hospices Civils de Lyon, Groupement Hospitalier Nord, Lyon cedex 4, France; Centre National de Référence France-Sud des Virus des Infections Respiratoires, Hospices Civils de Lyon, Groupement Hospitalier Nord, Lyon cedex 4, France; Laboratoire de Virologie, Institut des Agents Infectieux (IAI), Hospices Civils de Lyon, Groupement Hospitalier Nord, Lyon cedex 4, France; Université de Lyon, Université Claude Bernard Lyon 1 (UCBL1), Lyon, France; Université de Lyon, Virpath, CIRI, INSERM U1111, CNRS UMR5308, ENS Lyon, Université Claude Bernard Lyon 1, Lyon, France; Centre National de Référence France-Sud des Virus des Infections Respiratoires, Hospices Civils de Lyon, Groupement Hospitalier Nord, Lyon cedex 4, France; Laboratoire de Virologie, Institut des Agents Infectieux (IAI), Hospices Civils de Lyon, Groupement Hospitalier Nord, Lyon cedex 4, France; Université de Lyon, Université Claude Bernard Lyon 1 (UCBL1), Lyon, France; Université de Lyon, Virpath, CIRI, INSERM U1111, CNRS UMR5308, ENS Lyon, Université Claude Bernard Lyon 1, Lyon, France; Centre National de Référence France-Sud des Virus des Infections Respiratoires, Hospices Civils de Lyon, Groupement Hospitalier Nord, Lyon cedex 4, France; Laboratoire de Virologie, Institut des Agents Infectieux (IAI), Hospices Civils de Lyon, Groupement Hospitalier Nord, Lyon cedex 4, France; Université de Lyon, Université Claude Bernard Lyon 1 (UCBL1), Lyon, France; Université de Lyon, Virpath, CIRI, INSERM U1111, CNRS UMR5308, ENS Lyon, Université Claude Bernard Lyon 1, Lyon, France; Centre National de Référence France-Sud des Virus des Infections Respiratoires, Hospices Civils de Lyon, Groupement Hospitalier Nord, Lyon cedex 4, France

**Keywords:** whole-genome sequencing, next generation sequencing, SARS-CoV-2, COVID-19, genomic surveillance

## Abstract

Since the beginning of the COVID-19 outbreak, SARS-CoV-2 whole-genome sequencing (WGS) has been performed at unprecedented rate worldwide with the use of very diverse Next-Generation Sequencing (NGS) methods. Herein, we compare the performance of four NGS-based approaches for SARS-CoV-2 WGS. Twenty-four clinical respiratory samples with a large scale of Ct values (from 10.7 to 33.9) were sequenced with four methods. Three used Illumina sequencing: an in-house metagenomic NGS (mNGS) protocol and two newly commercialised kits including a hybridisation capture method developed by Illumina (DNA Prep with Enrichment kit and Respiratory Virus Oligo Panel, RVOP), and an amplicon sequencing method developed by Paragon Genomics (CleanPlex SARS-CoV-2 kit). We also evaluated the widely used amplicon sequencing protocol developed by ARTIC Network and combined with Oxford Nanopore Technologies (ONT) sequencing. All four methods yielded near-complete genomes (>99%) for high viral loads samples (*n* = 8), with mNGS and RVOP producing the most complete genomes. For mid viral loads (Ct 20–25), amplicon-based enrichment methods led to genome coverage >99 per cent for all samples while 1/8 sample sequenced with RVOP and 2/8 samples sequenced with mNGS had a genome coverage below 99 per cent. For low viral loads (Ct ≥25), amplicon-based enrichment methods were the most sensitive techniques. All methods were highly concordant in terms of identity in complete consensus sequence. Just one mismatch in three samples was observed in CleanPlex *vs* the other methods, due to the dedicated bioinformatics pipeline setting a high threshold to call SNP compared to reference sequence. Importantly, all methods correctly identified a newly observed 34nt-deletion in ORF6 but required specific bioinformatic validation for RVOP. Finally, as a major warning for targeted techniques, a loss of coverage in any given region of the genome should alert to a potential rearrangement or a SNP in primer-annealing or probe-hybridizing regions and would require further validation using unbiased metagenomic sequencing.

## 1. Introduction 

A novel human betacoronavirus, Severe Acute Respiratory Syndrome Coronavirus 2 (SARS-CoV-2), emerged in China in December 2019, rapidly spreading worldwide and resulting in the coronavirus disease 2019 (COVID-19) pandemic (Zhu et al. 2020). Whole-genome sequencing (WGS) of SARS-CoV-2 has played a major role since the onset of the pandemic. Notably WGS has contributed to design specific reverse-transcriptase polymerase chain reactions (RT-PCRs) (Corman et al. 2020), antiviral strategies (Dai et al. 2020), and vaccine candidates (Kames et al. 2020). SARS-CoV-2 WGS also allowed to explore lineage transmission and might be useful to assess the effectiveness of intervention measures (Grubaugh et al. 2019; Consortium et al. 2020; Oude Munnink et al. 2020; ‘Preliminary analysis of SARS-CoV-2 importation & establishment of UK transmission lineages,’ 2020). Furthermore SARS-CoV-2 genomic surveillance allowed the characterisation of some mutations for which the impact on virulence and transmissibility needs to be confirmed (Bal et al. 2020; Batty et al. 2020a; Holland et al. 2020). SARS-CoV-2 WGS is performed at an unprecedented rate worldwide with the use of very diverse Next Generation Sequencing (NGS) methods. Viral metagenomic NGS (mNGS) enabled early sequencing of the SARS-CoV-2 genome (Zhu et al. 2020) and is the method routinely used in the French National Reference Centre for Respiratory Viruses (NRC, Lyon, France). However, this method lacks sensitivity and cannot produce whole genomes for low viral load samples, except using a very high depth of sequencing (Bal et al. 2020). Targeted methods such as amplicon- or capture-based enrichments are promising candidates to overcome this issue. We proposed herein a formal comparative study of these different approaches to complete and enrich the sparse data already available (Xiao et al. 2020). Herein, we aimed to assess performance of four NGS-based approaches for SARS-CoV-2 WGS. Three used Illumina sequencing: an in-house metagenomic NGS (mNGS) protocol and two newly commercialised kits including a hybridisation capture method developed by Illumina (Respiratory Virus Oligo Panel, RVOP) and an amplicon sequencing method developed by Paragon Genomics (CleanPlex). We also assessed a widely used amplicon sequencing protocol developed by the ARTIC network and combined with Oxford Nanopore Technologies (ONT) sequencing (Consortium et al. 2020; https://artic.network/ncov-2019). The present evaluation covers experimental process as well as the associated bioinformatic solutions recommended for each technique. Importantly, this evaluation included two samples with a newly observed 34 nt-deletion in ORF6 (Quéromès et al. 2020) in order to assess the capacity to detect large modification in SARS-CoV-2 genome.

## 2. Methods

### 2.1. Sample selection

A total of twenty-four clinical samples (nasopharyngeal swab) with a broad range of representative SARS-CoV-2 cycle threshold (Ct) values (from 10.7 to 33.9) were selected for sequencing.

We defined three groups of samples: a low-Ct values group (Ct < 20), a medium-Ct values group (20≤Ct < 25), and high-Ct values group (Ct ≥ 25). Each sample was tested with a multiplexed real time RT-PCR targeting distinct RdRp gene regions namely IP4 and IP2 (Institut Pasteur assay, Paris, France [‘Molecular assays to diagnose COVID-19,’ n.d.]). In the present study, Ct values are those of the most sensitive target (IP4). Details of the Ct values for each sample are presented in the Supplementary Table S1. After nucleic acid extractions using the Easymag platform (bioMérieux, Lyon, France), several individual aliquots were performed for each extract and stored under the same conditions (frozen at −80°C). Thus, all extracts have been subjected to one freeze–thaw cycle whatever the methods of sequencing. Samples used in this study were collected as part of approved ongoing surveillance conducted by the NRC at the Hospices Civils de Lyon. The investigations were carried out in accordance with the General Data Protection Regulation (Regulation (EU) 2016/679 and Directive 95/46/EC) and the French data protection law (Law 78–17 on 06/01/1978 and Décret 2019–536 on 29/05/2019).

### 2.2. Sequencing approaches and technologies

#### 2.2.1. Illumina sequencing

##### 2.2.1.1. Viral metagenomics (mNGS)

DNase treatment (Life Technologies, Carlsbad, CA, USA) was performed after nucleic acid extraction in order to increase sensitivity of RNA virus detection and overcome human contamination (Bal et al. 2018). Nucleic acids were randomly amplified using the WTA2 kit (WTA2, Sigma-Aldrich, Darmstadt, Germany) and libraries were prepared using the Illumina Nextera XT kit (Illumina, San Diego, CA, USA). This in-house approach is routinely used to perform WGS SARS-CoV-2 genomic surveillance at the NRC (Bal et al. 2020; Danesh et al. 2020; Quéromès et al. 2020) for samples with a Ct value <20.

##### 2.2.1.2. Hybrid capture-based target enrichment

Hybrid capture-based approach was performed using the Illumina DNA Prep with Enrichment kit and Illumina RVOP V1.0. First, 15 µL of total nucleic acid were pre-treated with DNase following the same protocol as for mNGS (RNA concentration range from about 2 to 50 ng/µL, four samples below quantification limit). Then, double stranded cDNA synthesis was performed using 5 µL of DNase pre-treated RNA using the NEBNext^®^ Ultra™ II RNA First Strand Synthesis Module and NEBNext^®^ Ultra™ II Non-Directional RNA Second Strand kit (New England Biolabs, MA, USA). The obtained cDNA was used as input for library preparation with the Illumina DNA Prep with Enrichment kit according to manufacturer instructions (https://sapac.support.illumina.com/content/dam/illumina-support/documents/documentation/chemistry_documentation/illumina_prep/illumina-dna-prep-with-enrichment-reference-1000000048041-05.pdf). Pre-enriched libraries were pooled by mass and initial Ct values as recommended by the manufacturer. The enrichment was based on a hybridisation step with a respiratory virus biotinylated adjacent oligoprobes panel (O’Flaherty et al. 2018) recently expanded to include SARS-CoV-2. Enriched libraries were pooled before sequencing.

##### 2.2.1.3. Amplicon-based target enrichment

We implemented the CleanPlex SARS-CoV-2 panel (Paragon Genomics, Inc, Hayward, CA, USA) protocol for target enrichment and library preparation (https://www.paragongenomics.com/wp-content/uploads/2020/03/UG4001-01_-CleanPlex-SARS-CoV-2-Panel-User-Guide.pdf). Reverse transcription was performed using 11 μl of nucleic acid extract (the maximum volume recommended for the reaction). From 5 µL of reverse-transcribed RNA, multiplex PCR reactions were performed using 343 pairs of primers separated into two pools covering the entire genome of SARS-CoV-2 ranging from 116 bp to 196 bp, with a median size of 149 bp. Illumina indexes were introduced by PCR using twenty-five cycles. Following steps were performed according to the protocol. PCR products were finally pooled in equimolar ratios to reach the recommended final concentration of 4 nM.

For these three approaches, the prepared libraries were sequenced on an Illumina NextSeq^TM^ 550 with mid-output 2 × 150 bp flowcells for 26 h (mNGS and CleanPlex) or 2 × 75 bp flow cells for 15 h (RVOP).

### 2.2.2. ONT sequencing using amplicon-based target enrichment

We tested a multiplexed PCR amplicon approach derived from the ARTIC Network nCoV-2019 sequencing protocol (https://www.protocols.io/view/ncov-2019-sequencing-protocol-v2-bdp7i5rn) slightly modified by ONT (Oxford Nanopore Technologies, Oxford, UK) for better performance. We closely followed the PCR tiling of COVID-19 virus Nanopore protocol (Versions: PTC_9096_v109_revE_06Feb2020) published online https://community.nanoporetech.com/protocols/pcr-tiling-ncov/v/PTC_9096_v109_revF_06Feb2020. From 11 µL of extract, previously diluted according to Ct values (no dilution for Ct values higher than 18; 1:10 for Ct values between 15 and 18; 1:100 for Ct values lower than 15), a reverse-transcription was performed using SuperScript IV Reverse Transcriptase (SSIV, Invitrogen by Life Technologies, Carlsbad, USA) as well as non-specific primers: random hexamers and anchored polyT(23). Of note, based on SSIV supplier’s recommendations, modified incubate reactions were applied (annealing: 23°C, 10′; elongation: 55°C, 20′; inactivation: 80°C, 10′) instead of those specified in dedicated ONT protocol (annealing and elongation: 42°C, 50′; inactivation: 70°C, 10′). The multiplex PCR primers set v3, separated into two pools (A and B), was used to span the whole genome (https://artic.network/ncov-2019). For each pool, 2.5 µL of synthesised cDNA was used as template. Tiling 400 nt-amplicons with twenty base pair overlaps (not including primers) were generated, using thirty-five cycles for all samples. Samples were multiplexed by using the native barcode kits from ONT (EXP-NBD104 and EXP-NBD114). The library was prepared using the SQK-LSK109 kit. A total amount of 15 ng of this final prepared library was sequenced on a FLO-MIN106 (R9.4.1) flow cell, multiplexing twenty-four samples per run. ONT sequencing was run until the exhaustion of nanopores (≈72 h).

### 2.3. Sequencing data analysis

mNGS data were analysed with an in-house pipeline. Briefly, low quality (<Q30), human reads and reads shorter than fifty nucleotides in length were filtered out. Remaining reads were aligned to the SARS-CoV-2 reference genome (isolate Wuhan-Hu-1, EPI_ISL_402125, MN908947) using the BWA-MEM (v0.7.15-r1140) algorithm (Li and Durbin 2009). Consensus sequences were generated through a simple majority rule using custom perl script. These sequences were used as the patients’ own mapping reference for further realignment of the reads. Final consensus sequence was called at 10× using no-clip alignment. This in-house pipeline is publicly available online, https://github.com/jossetlab/WTA_SARS-CoV2_Pipeline. Visual inspection of read alignments was performed using Integrative Genomics Viewer (IGV) (Robinson et al. 2011) around regions with large drop in coverage to detect and define potential deletions. For the CleanPlex SARS-CoV-2 amplicon approach, data analysis was performed according to the supplier's recommendations with a pipeline developed by Sophia Genetics (V1), including a primers trimming step (https://www.paragongenomics.com/wp-content/uploads/2020/03/UG4001-01_-CleanPlex-SARS-CoV-2-Panel-User-Guide.pdf). Sequencing data from the RVOP capture approach were parsed using the Illumina Dynamic Read Analysis for GENomics (DRAGEN; v3.5.13; Illumina) Bio-IT Platform. Finally, ONT sequencing data were analysed by implementing the recommended bioinformatics developed by ARTIC and available online (v1.1.0) (https://artic.network/ncov-2019/ncov2019-bioinformatics-sop.html). Briefly, after the run was done, basecalling was performed using Guppy high accuracy models (v3.5.2). As chimeric reads are the predominant source of cross barcode assignment errors (Xu et al. 2018), we followed the ARTIC recommendations and demultiplex using strict Guppy_barcoder parameters to ensure barcodes are present at each end of the fragment. ARTIC pipeline was run using Minimap2 (v 2.17) for alignment (Li 2018) and nanopolish (v0.13.2) for variant calling (Loman, Quick, and Simpson 2015). To overcome the 400x depth limitation of ARTIC bioinformatics pipeline, we independently generated coverage plot from BEDtools (v2.29.2) data (Quinlan and Hall 2010), after Minimap2 alignment of the output filtered FASTQ files generated by the guppyplex command of ARTIC bioinformatics pipeline. Five parameters were specifically evaluated and compared: coverage metrics such as depth and breadth of coverage at 10× for Illumina sequencing data and 20× for ONT sequencing data, number of SNPs between methods after pairwise alignment of consensus sequences, and detection of a particular 34 nt-deletion detected with our in-house reference mNGS method. Of note, 20× minimum depth threshold has been chosen to evaluate ARTIC-ONT protocol as it is the default setting to call the consensus in the standard ARTIC bioinformatics pipeline. It enables to overcome potential error rate issue and resolve the vast majority of ‘simple substitutions’. The proportion (%) of complete genome coverage are presented as medians with interquartile ranges [IQR] and compared using the non-parametric Friedman test.

## 3. Results

### 3.1. Sensitivity assessment of four SARS-CoV-2 sequencing approaches

Four widely used NGS methods were evaluated: an unbiased mNGS approach, and three targeted approaches based on hybridisation capture (RVOP) or amplicon sequencing (CleanPlex and ARTIC-ONT). For high viral loads (Ct <20), the four sequencing approaches yielded almost-complete genome (>99% covered at 10× for Illumina and 20× for ONT sequencing) for all samples. A significant difference in genome coverage distribution (*P* < 0.001) was found; the highest median of coverage was for RVOP and mNGS (Table 1). For mid-Ct samples, one sample sequenced with RVOP and two samples sequenced with mNGS had a genome coverage below 99 per cent (93.4% with RVOP, 72.6% and 56.5% with mNGS, respectively).

**Table 1.** Proportion of coverage throughout the complete SARS-CoV-2 genome at 10× for the three Illumina sequencing methods and at 20× for the ARTIC-ONT sequencing method.

		% Coverage				
Ct group	Sample_Ct	RVOP	CleanPlex	ARTIC	mNGS	*P*
				-ONT		
Low (Ct < 20)	1_10.7	100.0	99.2	99.6	100.0	
	2_14.5	100.0	99.5	99.6	99.9	
	3_16.4	99.9	99.7	99.6	99.8	
	4_16.6	99.9	99.5	99.6	99.8	
	5_16.7	99.9	99.5	99.6	99.6	
	6_17.0	99.9	99.7	99.6	99.8	
	7_17.6	99.8	99.4	99.5	99.7	
	8_17.7	99.8	99.3	99.5	99.5	
	Median	99.9	99.5	99.6	99.8	
	[IQR]	[99.9–99.9]	[99.4–99.6]	[99.6–99.6]	[99.9–99.9]	<0.001
Mid (20≤Ct < 25)	9_20.0	99.8	99.5	99.6	99.7	
	10_20.4	99.8	99.7	99.6	99.6	
	11_21.0	99.8	99.7	99.6	99.7	
	12_21.3	99.9	99.2	99.6	99.8	
	13_21.6	99.8	99.7	99.6	99.9	
	14_22.9	99.9	99.6	99.6	56.5	
	15_24.3	99.7	99.4	99.6	72.6	
	16_24.6	93.4	99.0	99.6	99.6	
	Median	99.8	99.6	99.6	99.7	
	[IQR]	[99.8–99.8]	[99.4–99.7]	[99.6–99.6]	[92.9–99.7]	0.06
High (Ct ≥ 25)	17_25.0	91.9	99.2	99.6	99.1	
	18_25.7	92	99.7	99.6	88.6	
	19_27.4	99.8	99.3	99.6	7.0	
	20_28.1	99.8	99.7	99.6	90.2	
	21_29.9	99.7	99.5	99.6	0.0	
	22_32.4	55.2	99.5	95.0	10.6	
	23_33.0	2.2	95.9	72.5	0.7	
	24_33.9	38.3	99.0	98.6	0.0	
	Median	92.0	99.4	99.6	8.8	
	[IQR]	[51.0–99.8]	[99.2–99.6]	[97.7–99.6]	[0.5–89.0]	0.005

Cycles threshold (Ct) values were determined using the most sensitive target of the RdRp Institute Pasteur RT-PCR assay (IP4). Using an R script, these proportions (%) were calculated from depth files generated by BEDtools from output aligned bam files of each specific-method pipeline. The percentage of genome coverage are presented as medians with interquartile ranges [IQR] and compared using the non-parametric Friedman test.

Among samples with low viral loads (Ct ≥25), significant discrepancies in genome coverage were observed between methods (*P* = 0.005); 8.8 per cent median coverage in samples sequenced with mNGS, 92.0 per cent for RVOP, and highest median coverage were obtained with amplicon sequencing (99.4%, CleanPlex; 99.6%, ARTIC-ONT).

While RVOP and mNGS enabled to generate the complete genome (maximum coverage was 100% for highest viral loads), amplicon-based target enrichment did not allow to cover SARS-CoV-2 genome ends, as expected by considering the design of ARTIC-ONT and CleanPlex protocols. The highest coverage was 99.6 per cent and 99.7 per cent for ARTIC-ONT and CleanPlex, respectively.

**Figure 1.**
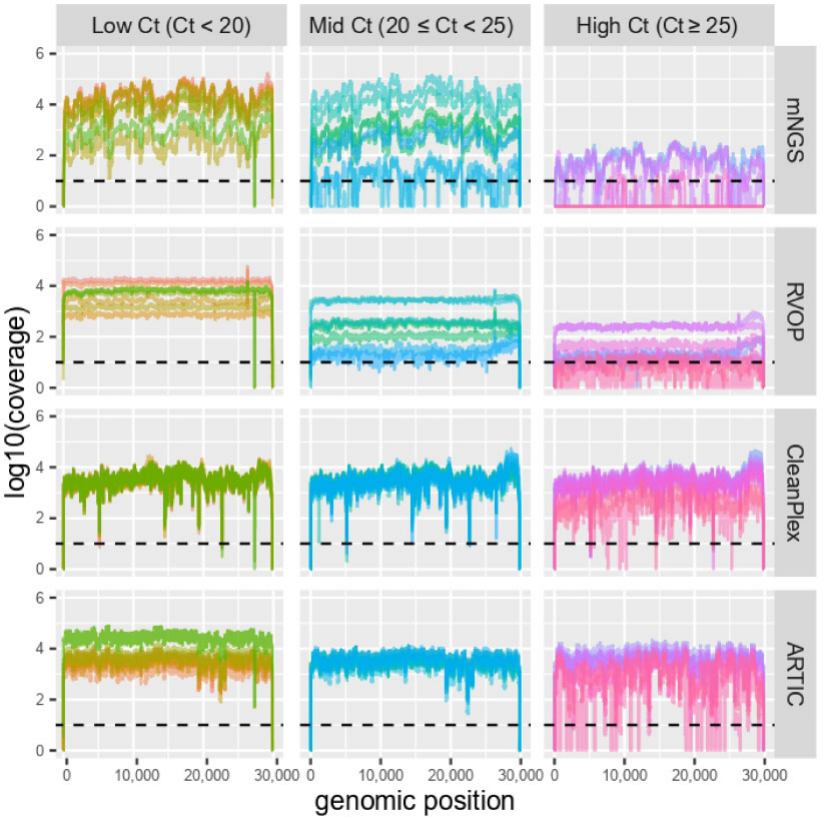
Plots of coverage according to evaluated methods and Cycle threshold (Ct) values groups. Dotted lines indicate the minimum depth of 10× for Illumina methods and 20× for ONT method. Missing sites in the genome are those with a coverage <10× for Illumina methods and <20× for the ARTIC-ONT method. Using an R script, these plots were constructed via ggplot2 from depth files generated by BEDtools from output aligned bam files of each specific-method pipeline.

Irrespective of Ct value, RVOP provided an even depth of coverage throughout the genome, while uneven depth of coverage was obtained with mNGS and marked drops in sequencing depth were observed for CleanPlex (Fig. 1). These drops corresponded to amplicons that were poorly amplified: from 5,159 to 5,199 (drop # 1), from 14,430 to 14,505 (drop # 2), from 19,337 to 19,399 (drop # 3), and from 22,641 to 22,715 (drop # 4; Fig. 1). Regarding low- and mid-Ct samples, 1, 3, and 8 had a coverage <10× at drops # 2, #1, and # 4, respectively. Evenness of depth of coverage was intermediate for ARTIC for low- and mid-Ct values, while high-Ct values yielded more uneven depth.

### 3.2. Comparison of complete genome consensus sequence

**Figure 2.**
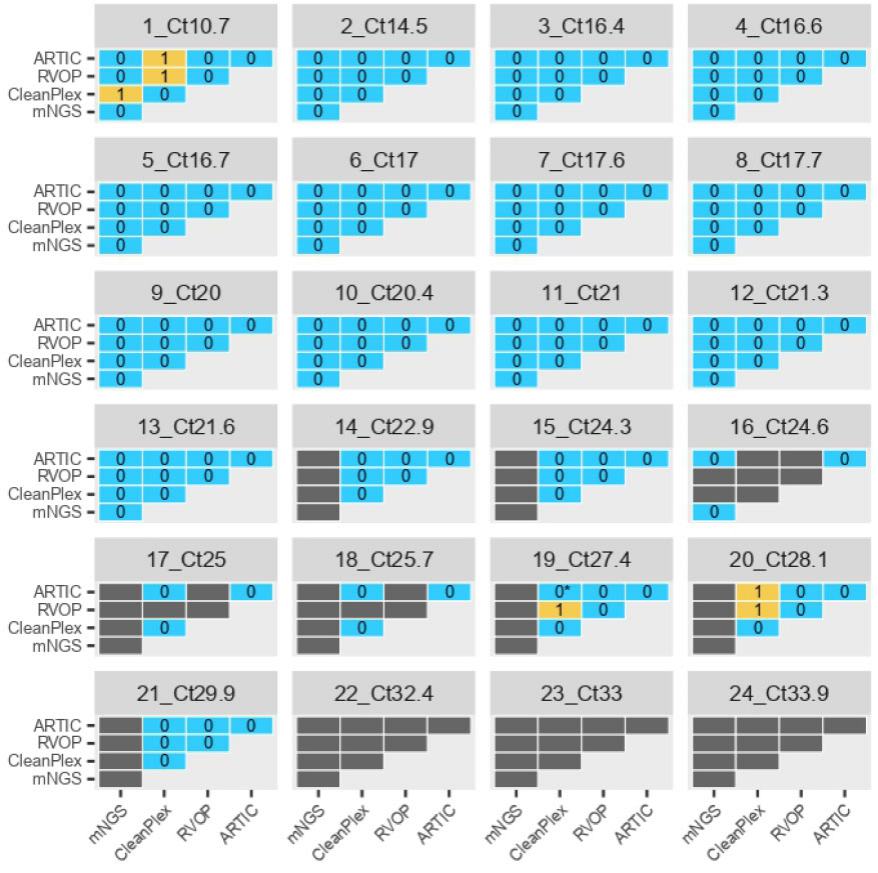
Mismatch count between consensus sequences generated by each method compared two by two for each sample. These matrices were generated only from consensus with determined bases for more than 99 per cent of the genome. If one sequence of the two had more than 1 per cent of undetermined bases (N), comparison was not assessed, grey tiles. Blue tiles correspond to perfect identity and orange tile correspond to mismatches, the number of mismatches is indicated inside the tile. Matrices were generated with an R script using Decipher (alignment), ape (distance matrices), and ggplot2 (charts) libraries. Of note, undetermined bases and deletions were not considered in the calculation of mismatches. * For the sample #19: the position 533 is undetermined by ARTIC method, and therefore no SNP is observed between ARTIC and CleanPlex methods.

Almost-complete genomes generated with the four methods were further compared to define accuracy of each approach (Fig. 2). Of note, nine, seven, and one samples sequenced, respectively, with mNGS, RVOP and CleanPlex were excluded from the analysis due to a coverage <99 per cent at 10×, *vs* three samples for ARTIC-ONT protocol at 20×. For the other samples, all consensus sequences were strictly identical, expect for three samples for which CleanPlex led to one mismatch compared to the other methods (sample # 1, position 28086; sample # 19, position 533; sample # 20, position 11083). By analysing VCF files, these three positions corresponded to SNPs (G280086T, G533T, G11083T) with variant frequency <70 per cent which is the threshold set for calling a SNP in Sophia Genetics pipeline while other pipelines set this threshold at 50 per cent. No additional discrepancies were observed for a minimum of 90 per cent coverage (Supplementary Fig. S1) as well as a minimum of 70 per cent coverage (Supplementary Fig. S2). Thus, genomes with coverage between 70 per cent and 99 per cent are not more likely to have sequencing errors.

### 3.3. Performance of the methods for the detection of a large deletion in SARS-CoV-2 genome

A 34 nt-deletion in the ORF6 at loci 27266 was detected in two samples (samples # 7 and # 8) by all the evaluated approaches both in alignment files and consensus sequences. For RVOP, it is noteworthy that DRAGEN output alignment files showed an absence of coverage in the region but there were no spanning reads to visualise the breakpoint of the deletion. Similar results were observed using BWA-MEM to align the reads. However, DRAGEN output VCF clearly highlighted the 34 nt-deletion as well as the final consensus sequence for both samples. Using Minimap2 as aligner, the deletion was clearly identified with reads spanning the breakpoint of the deletion on the reference genome (Supplementary Fig. S3).

## 4. Discussion

We present herein the evaluation of four representative NGS-based approaches for SARS-CoV-2 whole genome characterisation. Unbiased mNGS is the most appropriate method to identify novel emerging pathogens such as SARS-CoV-2 (Mokili, Rohwer, and Dutilh 2012; Zhu et al. 2020) but did not produce all WGS for mid- and low-Ct values samples in the present study. In contrast, the three targeted methods evaluated herein exhibited higher sensitivity compared to mNGS by reaching a high proportion of the genome covered. In particular, amplicon-based target enrichment using CleanPlex or ARTIC-ONT was the most sensitive approach and allowed WGS of samples with Ct values up to 33.9 in this present study. Although not reported herein, amplicon-based target enrichment can be impacted by SNP or indels located within primer-annealing regions even if their tiling amplicon designs aims to reduce the impact of such modifications. As SARS-CoV-2 may potentially evolve, primers for amplicon-based target enrichment need to be constantly updated. Such updates will be important for CleanPlex as we have observed coverage dropout issues in four regions of the genome irrespective of Ct value, suggesting potential mismatches in primer-annealing regions that may lead to variation in the optimal annealing temperature and thus to a decrease in the amplification efficiency.

All four methods were highly concordant in terms of identity in complete consensus sequence. Just one mismatch in three samples was observed in CleanPlex *vs* the other methods, due to the dedicated pipeline setting a high threshold to call SNP compared to reference genome. Considering the very low evolution rate of this virus that can be explained by the editing function of its polymerase, just one substitution can have high repercussions in terms of evolutionary assessment (Worobey et al. 2020).

Furthermore, a novel 34 nt-deletion in the ORF6 previously observed with our in-house mNGS method, was detected by the two evaluated tiling amplicon sequencing methods (ARTIC and CleanPlex). However, larger deletions, such as the 382-nucletide deletion in the ORF7b and ORF8 previously described (Su et al. 2020), spanning primer-annealing regions, would be difficult to define using amplicon-based target enrichment methods. Missing regions with no coverage should be carefully investigated using other methods. The 34 nt-deletion was also detected using RVOP despite an adjacent oligoprobes design according to which oligoprobes are placed end-to-end to cover the entire genome without overlapping as in a tiling design.

The present study does have several limitations. First, the four methods were tested on a limited number of European samples, all affiliated to the lineage B1, according to the Pangolin classification (Rambaut et al. 2020); the present evaluation should be confirmed in larger studies including other lineages in order to be more representative of the global ecology of this emerging virus. As SARS-CoV-2 sequencing is an expanding market and other methods have been published (Batty et al. 2020b; Freed et al. 2020; Paden et al. 2020) or are probably under development, the present study is not exhaustive. However, the approaches selected herein are representative of the methods mostly used during pandemics and are easy to implement in a diagnostic laboratory.

Another critical point is the absence of accurate cross-contamination evaluation. Negative controls, starting from the extraction, were included in every run for all methods except for the CleanPlex due to a lack of reagents. However, as all negative controls sequenced with the three evaluated methods (mNGS, ARTIC-ONT, and RVOP) indicated very limited cross-contamination (<31 reads mapping SARS-CoV-2), and considering the congruence between consensus sequences generated with the different methods, we estimate that there was also no cross-contamination during the CleanPlex run. As cross-contamination risk increases with the sample size, this technical point needs to be evaluated in larger batches in further and future investigations. Another point of criticism in the present study is that dedicated bioinformatic processes have been implemented as recommended by suppliers. Except for RVOP for which we compared the aligner using in DRAGEN and minimap2, we chose not to perform a comparison of the bioinformatic tools. In this context, further bioinformatic evaluations are needed especially concerning parameters such as the variant frequency threshold for calling SNP compared to reference sequence, as well as the minimum depth for calling consensus. Importantly, both dedicated pipelines implemented for amplicon-based approach analysis include a primer trimming process that is mandatory to avoid calling the reference bases instead of potential SNP within primer-annealing regions. As previously suggested, such errors may impair phylogeny analysis (Worobey et al. 2020). Additionally, *de novo* assembly should be evaluated as it may provide better assessment of rearrangements such as large deletions, insertions, and duplications that can be missed by classical alignment analyses, but this method is time-consuming and may not be appropriate for genomes with low coverage. More broadly, a repeatability test is mandatory to support results obtained from the present evaluation. Finally, only one sample type or storage condition has been tested. Different quality samples should be tested to assess if any of the methods are more adapted to different sample types or conditions.

Cost effectiveness and turnaround time were difficult to assess. Indeed, many parameters such as laboratory equipment and prices negotiated with suppliers may affect results. However, a few general comments can be made. In the present study, twenty-four samples were multiplexed for Illumina sequencing which is not optimal in terms of cost effectiveness; larger batches would contribute to reduce the cost of sequencing. Nevertheless, the impact of a high multiplexing on the sequencing depth and cross-sample contamination needs to be carefully evaluated. Conversely, ONT sequencing is a cost-effective method for small batches of samples. Of note, the MinION run-time of 72 h herein (*vs* 15 h for the fastest Illumina kit used*)* could have been decreased to a few hours but fast sequencing was not the objective of the present study; we chose to sequence until total nanopore exhaustion (72 h) in order to obtain the greatest depths. In addition, a major point is that ONT sequencers are small devices, virtually maintenance-free, and affordable for small laboratories. Concerning the library preparation, a two-day turnaround was needed for the four methods evaluated. However, RVOP and mNGS are the most tedious ones and require well-trained staff.

To conclude, the data presented herein are useful for clinical and research teams who want to implement SARS-CoV-2 sequencing and chose the most suitable protocol according to the application. To summarise, mNGS remains the gold standard for samples with high viral load to obtain a maximum of information without any bias. For low- and mid-Ct values, RVOP leads to very high coverage as well, enabling genome end sequencing, contrary to amplicon methods. For higher Ct values, amplicon-based enrichment is a very interesting alternative, in particular ARTIC-ONT protocol that did not show any major dropout issues in this present evaluation. However, as a reminder, loss of coverage in any given region of the genome should alert to a potential rearrangement or an SNP in primer-annealing or probe-hybridizing regions and would require further validation using unbiased metagenomic sequencing.

## Supplementary data

Supplementary data are available at *Virus Evolution* online.

## Supplementary Material

veaa075_Supplementary_Data
