# Supplementary material for: Evaluation of NGS-based approaches for SARS-CoV-2 whole genome characterisation
Source: Virus Evol. 2020 Oct 5;6(2):veaa075. doi: 10.1093/ve/veaa075 (PMC7665770; doi:10.1093/ve/veaa075)
Supplement: veaa075_Supplementary_Data [file veaa075_supplementary_data.zip › suppl_data/Revised_Supplementarytable1.pdf]

Sample ID	Ct (IP4)	Ct (IP2)
1	10.7	11.6
2	14.5	15.4
3	16.4	17.0
4	16.6	17.1
5	16.7	17.4
6	17.0	18.0
7	17.6	18.4
8	17.7	18.4
9	20.0	20.7
10	20.4	21.0
11	21.0	21.9
12	21.3	22.0
13	21.6	22.7
14	22.9	23.5
15	24.3	24.8
16	24.6	25.4
17	25.0	25.4
18	25.7	26.6
19	27.4	28.3
20	28.1	28.9
21	29.9	30.8
22	32.4	33.9
23	33.0	36.0
24	33.9	35.3
