# Supplementary figures and images for: Evaluation of NGS-based approaches for SARS-CoV-2 whole genome characterisation
Source: Virus Evol. 2020 Oct 5;6(2):veaa075. doi: 10.1093/ve/veaa075 (PMC7665770; doi:10.1093/ve/veaa075)

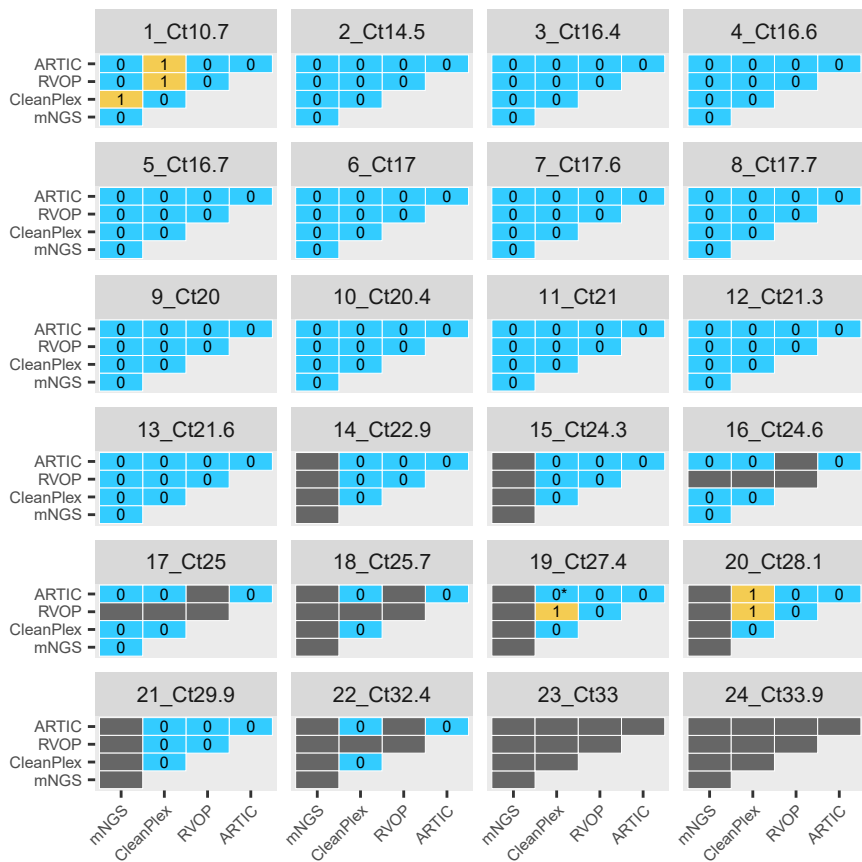

Supplement: veaa075_Supplementary_Data [file veaa075_supplementary_data.zip › suppl_data/Revised_Supplementary_Figure1.pdf]

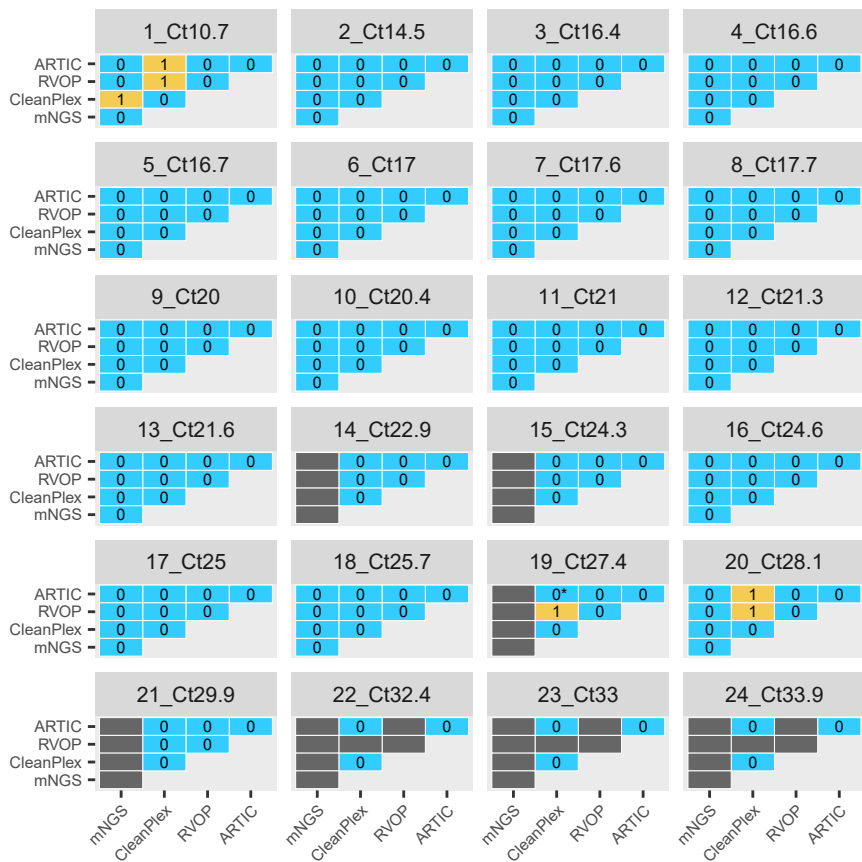

Supplement: veaa075_Supplementary_Data [file veaa075_supplementary_data.zip › suppl_data/Revised_Supplementary_Figure2.pdf]

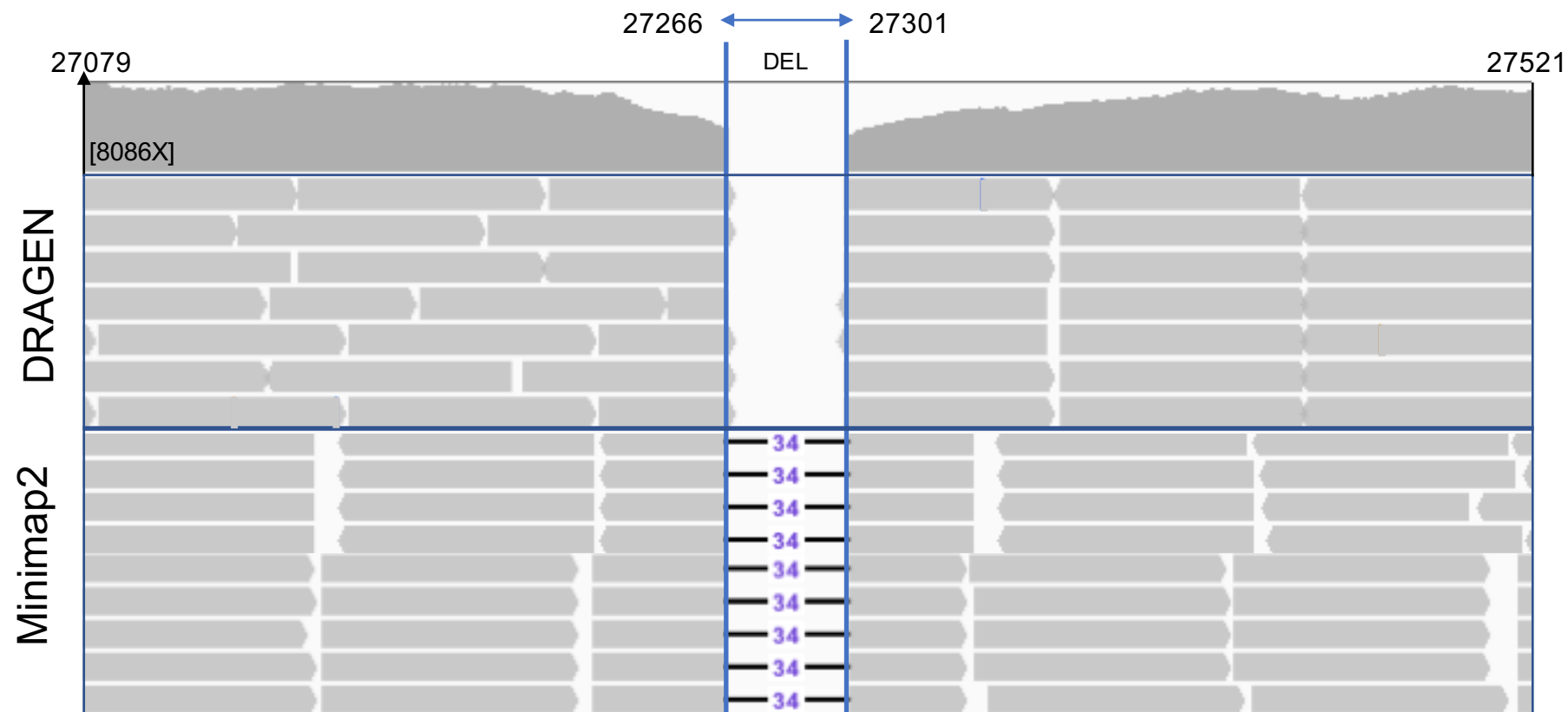

Supplement: veaa075_Supplementary_Data [file veaa075_supplementary_data.zip › suppl_data/Revised_Supplmentary_Figure3.pdf]
